# Supplementary material for: Is refugee experience in childhood a risk for poorer health in adulthood?—A Swedish national survey study
Source: PLOS Glob Public Health. 2023 Nov 8;3(11):e0002433. doi: 10.1371/journal.pgph.0002433 (PMC10631682; doi:10.1371/journal.pgph.0002433)
Supplement: S1 Text — Table A in S1 Text: A detailed description of the variables in the Swedish National Public Health Survey ‘Health on equal terms’ (2018–20) and the variables that we analysed in this study. Table B in S1 Text: How we identified migrant and refugee experience in childhood among participants in the National Public Health Survey in 2018 and 2020. The strategy outlined was developed in consultation with Swedish Board of Migration. Table C in S1 Text: Proportions of country of origin, sex and time in Sweden among those with a refugee experience in childhood, those with a refugee experience in childhood, or Swedish-born. Stratified by age groups. The numbers are unweighted counts (n) and unweighted proportions (%). Table D in S1 Text. Development of the model. Stratified by sex. Table E in S1 Text. Development of the model. Stratified by sex. Fig A S1 in Text: Illustration of geographical origin (unweighted), time of conflicts, and age groups1. (DOCX) [file pgph.0002433.s002.docx]

Supplementary Material

Is refugee experience in childhood a risk for poorer health in adulthood - A Swedish national survey study

**Authors:**Erica Mattelin, Amal R Khanolkar, Laura Korhonen, Jill W Ahs and Frida Fröberg

**Table A: A detailed description of the variables in the Swedish National Public Health Survey ‘Health on equal terms’ (2018-20) and the variables that we analysed in this study.**

| **Indicator and outcome variables** | **As originally assessed in the national public health survey** | **Response options** | **Variables and response options in analyses in this study** |
| --- | --- | --- | --- |
| Sexual identity | *How would you define your sexual identity?* | 1 heterosexual  2 bisexual  3 homosexual  4 other  5 don’t know | 1 Heterosexual  2-3 Sexual minority  4-5 Excluded if not “yes” on gender identity |
| Gender identity | *Are you or have you been a transgender person?* | 1 yes  2 no  3 don’t know | 1 Sexual minority  3 Excluded if not sexual minority on sexual identity |
| Psychological distress | For 2020, Kessler-6  *In the past month, how often did you feel*   1. *nervous?* 2. *Hopeless* 3. *Restless or fidgety?* 4. *so depressed that nothing could cheer you up?* 5. *that everything was an effort?* 6. *worthless?* | 4 All the time  3 Most of the time  2 Some of the time  1 A little of the time  0 None of the time | Total score: 0-12 No mental ill-health |
|  |  |  | 13-24 Mental ill-health |
|  | For 2018, General Health Questionnaire (GHQ) -5  *Over the past few weeks:*   1. *have you felt constantly unhappy and depressed?* 2. *have you been losing confidence in yourself?* 3. *have you felt constantly tense?* 4. *have you thought of yourself as a worthless person?* 5. *have you felt that you couldn’t overcome your difficulties?* | 0 Not at all  0 No more than usual  1 Rather more than usual  1 Much more than usual | Score: 2< mental ill-health  2> No mental ill-health |
| Suicidal ideation | *Have you ever been in a situation where you seriously considered taking your own life?* | 1 No  2 Yes, during the past 12 months  3 Yes, more than a year ago | 1 No  2-3 Yes |
| Suicide attempts | *Have you ever attempted to take your own life?”.* | 1 No  2 Yes, during the past 12 months  3 Yes, more than a year ago | 1 No  2-3 Yes |
| General health | *How would you rate your general health?* | 1 Very good  2 Good  3 Moderate  4 Bad  5 Very bad | 1-2 Very good or good  3 Moderate  4-5 Bad or very bad |
| At-risk alcohol consumption | Alcohol Use Disorders Identification Test | Total sum score of 0–12 | <5 risk consumption for women |
|  |  |  | <6 risk consumption for men |
| Substance use | *Have you ever used cannabis (e.g., hashish or marijuana)?* | 1 No  2 Yes, more than 12 months ago  3 Yes, in the past 12 months  4 Yes, in the past 30 days | 1 No  2-4 Yes |
|  | *Have you ever used an illicit drug other than cannabis (e.g. amphetamine, cocaine, heroin, ecstasy or LSD)?* |  |  |

**Table B: How we identified migrant and refugee experience in childhood among participants in the National Public Health Survey in 2018 and 2020. The strategy outlined was developed in consultation with Swedish Board of Migration.**

| Participants were categorized as refugees if they met the criteria for A *AND* B *AND* C | A They were born outside of Sweden (Country of birth -  The Total Population Register) |
| --- | --- |
|  | B Migrated during 1939-1945 regardless of country (Year of Immigration – the Total Population Register) OR categorized as a refugee according to the Swedish Migration Board (Categorized as refugee (FlyKat)– STATIV) OR Immigrated from Former Yugoslavia between 1980 - Onwards (Country of birth+ InvAr – The Total Population Register) OR Immigrated from Iran before 2007 (Country of birth + Year of Immigration– The Total Population Register) OR Immigrated from Syria, Eritrea, Afghanistan, Somalia (Country of birth– The Total Population Register) OR Immigrated from the Soviet Union until 2010 (Country of birth – The Total Population Register) OR Immigrated from the Iraq until 2010 (Country of birth – The Total Population Register)  C Migrated before the age of 18 (Year of Birth - Year of Immigration – the Register of the Total Population) |
| Participants were categorized as migrants if they met the criteria for D *AND* E *AND* F | D They were born outside of Sweden (Country of Birth -  The Total Population Register) |
|  | E Immigrated from Former Yugoslavia before 1980 (Country of birth + Year of Immigration – The Total Population Register) OR Immigrated from Iran after 2007 (Country of birth + Year of Immigration– The Total Population Register) OR Migrated from South America, Asia, Africa (Country of birth – The Total Population Register) OR Europe but not EU after 1945 (Country of birth – The Total Population Register) OR Immigrated from the Iraq after 2010 (Country of birth – The Total Population Register) OR Immigrated from the Soviet Union after 2010 (Country of birth – The Total Population Register) OR Oceania OR North America OR They were born in the Nordic Countries OR Finland OR EU28 after 1945 (Year of Birth – Year of Immigration – the Register of the Total Population).  F Migrated before the age of 18 (Year of Birth - Year of Immigration – the Register of the Total Population) |
| Participants were categorized as Swedish-born if they met the criteria G | G They were born in Sweden |

**Table C: Proportions of country of origin, sex and time in Sweden among those with a refugee experience in childhood, those with a refugee experience in childhood, or Swedish-born. Stratified by age groups. The numbers are unweighted counts (n) and unweighted proportions (%).**

|  | Swedish-born | | | | Migrant experience in childhood | | | | Refugee experience in childhood | | | | |
| --- | --- | --- | --- | --- | --- | --- | --- | --- | --- | --- | --- | --- | --- |
|  | 18–25 | | 26–64 | | 18–25 | | 26–64 | | | 18–25 | | 26–64 | |
|  | 10911 | | 74579 | | 601 | | 2135 | | | 617 | | 572 | |
|  | N | % | N | % | N | % | N | % | | N | % | N | % |
| Time in Sweden |  |  |  |  |  |  |  |  | |  |  |  |  |
| *<2* |  |  |  |  | 26 | 4.3 | 0 | 0 | | 52 | 8.7 | 0 | 0 |
| *2-6* |  |  |  |  | 64 | 10.7 | 0 | 0 | | 224 | 37.3 | 0 | 0 |
| *6-10* |  |  |  |  | 126 | 21.0 | <5 | 0 | | 141 | 23.5 | <5 | 0.7 |
| *>10* |  |  |  |  | 383 | 63.9 | 2134 | 100 | | 184 | 30.6 | 566 | 99.3 |
| Sex |  |  |  |  |  |  |  |  | |  |  |  |  |
| *Females* | 6285 | 57.6 | 41368 | 55.5 | 358 | 59.6 | 1210 | 56.7 | | 296 | 48 | 291 | 50.9 |
| Countries of origin |  |  |  |  |  |  |  |  | |  |  |  |  |
| *Afghanistan* |  |  |  |  | 0 | 0 | 0 | 0 | | 87 | 14.1 | 13 | 2.3 |
| *Africa* |  |  |  |  | 44 | 7.3 | 59 | 2.8 | | 24 | 3.9 | 9 | 1.6 |
| *Asia* |  |  |  |  | 218 | 36.3 | 437 | 20.5 | | 59 | 9.6 | 8 | 1.4 |
| *Eritrea* |  |  |  |  | 0 | 0 | 0 | 0 | | 49 | 7.9 | 10 | 1.7 |
| *EU28 excepts the Nordics* |  |  |  |  | 176 | 29.3 | 409 | 19.2 | | <5 | 0.5 | <5 | 0.3 |
| *Europe without EU28 and the Nordics* |  |  |  |  | 45 | 7.5 | 114 | 5.3 | | 17 | 2.8 | 8 | 1.4 |
| *Finland* |  |  |  |  | 11 | 1.8 | 601 | 28.1 | | 0 | 0 | 0 | 0 |
| *Former Yugoslavia* |  |  |  |  | 0 | 0 | 93 | 4.4 | | 44 | 7.1 | 272 | 47.6 |
| *Iraq and Iran* |  |  |  |  | 11 | 1.8 | 0 | 0 | | 112 | 18.1 | 184 | 32.2 |
| *North America* |  |  |  |  | 19 | 3.2 | 60 | 2.8 | | <5 | 0.2 | 0 | 0 |
| *The Nordics except Sweden* |  |  |  |  | 42 | 7.0 | 165 | 7.7 | | <5 | 0.2 | <5 | 0.2 |
| *Oceania* |  |  |  |  | <5 | 0.7 | 9 | 0.4 | |  |  |  |  |
| *Somalia* |  |  |  |  | 0 | 0 | 0 | 0 | | 89 | 14.4 | 18 | 3.1 |
| *Former Soviet Union* |  |  |  |  | 0 | 0 | 0 | 0 | | 0 | 0 | 12 | 2.1 |
| *Sweden* | 10911 | 100 | 74579 | 100 | 0 | 0 | 0 | 0 | | 14 | 2.3 | <5 | 0.3 |
| *South America* |  |  |  |  | 31 | 5.2 | 188 | 8.8 | | <5 | 0.5 | <5 | 0.2 |
| *Syria* |  |  |  |  | 0 | 0 | 0 | 0 | | 113 | 18.3 | 32 | 5.6 |

**Table D. Development of the model. Stratified by sex.**

| Females | Mode1 1: Unadjusted | | Model 2: Adjusted for age | | Model 3: Adjusted for sexual minority status (SM) | | Final model: Adjusted for age and SM. | |
| --- | --- | --- | --- | --- | --- | --- | --- | --- |
|  | Refugee experience in childhood | Migrant experience in childhood | Refugee experience in childhood | Migrant experience in childhood | Refugee experience in childhood | Migrant experience in childhood | Refugee experience in childhood | Migrant experience in childhood |
| General Health | 0.97 (0.56-1.68) | 1.19 (0.85-1.66) | 1.13 (0.65-1.98) | 1.23 (0.88-1.72) | 0.93 (0.51-1.69) | 1.15 (0.81-1.65) | 1.15 (0.62-2.12) | 1.21 (0.84-1.72) |
| Psychological distress | 1.57 (1.12-2.19) | 1.47 (1.17-1.84) | 1.07 (0.76-1.51) | 1.34 (1.07-1.68) | 1.47 (1.03-2.09) | 1.39 (1.21-1.74) | 1.08 (0.75-1.53) | 1.30 (1.04-1.63) |
| Suicide thoughts | 0.72 (0.49-1.07) | 1.28 (1.03-1.60) | 0.48 (0.32-0.72) | 1.17 (0.93-1.46) | 0.62 (0.40-0.97) | 1.18 (0.95-1.48) | 0.47 (0.30-0.72) | 1.11 (0.89-1.39) |
| Suicide attempts | 1.21 (0.69-2.12) | 1.45 (1.04-2.01) | 0.87 (0.49-1.54) | 1.34 (0.96-1.86) | 1.12 (0.60-2.10) | 1.30 (0.92-1.83) | 0.94 (0.51-1.74) | 1.24 (0.88-1.76) |
| At-risk alcohol use | 0.18 (0.09-0.33) | 0.73 (0.57-0.95) | 0.14 (0.08-0.27) | 0.69 (0.54-0.90) | 0.19 (0.10-0.35) | 0.74 (0.57-0.96) | 0.15 (0.08-0.29) | 0.71 (0.54-0.92) |
| Substance use | 1.03 (0.50-2.14) | 1.82 (1.17-2.81) | 0.45 (0.21-0.95) | 1.42 (0.90-2.23) | 0.93 (0.43-2.02) | 1.59 (1.01-2.50) | 0.47 (0.22-1.03) | 1.31 (0.82-2.10) |

**Table E. Development of the model. Stratified by sex.**

| Males | Mode1 1: Unadjusted | | Model 2: Adjusted for age | | Model 3: Adjusted for sexual minority status (SM) | | Final model: Adjusted for age and SM. | |
| --- | --- | --- | --- | --- | --- | --- | --- | --- |
|  | Refugee experience in childhood | Migrant experience in childhood | Refugee experience in childhood | Migrant experience in childhood | Refugee experience in childhood | Migrant experience in childhood | Refugee experience in childhood | Migrant experience in childhood |
| General Health | 0.53 (0.28-1.02) | 1.62 (1.05-2.49) | 0.68 (0.35-1.32) | 1.67 (1.09-2.58) | 0.48 (0.23-1.00) | 1.58 (1.01-2.46) | 0.61 (0.29-1.29) | 1.62 (1.04-1.54) |
| Psychological distress | 1.44 (0.99-2.11) | 1.29 (0.95-1.75) | 1.05 (0.71­-1.55) | 1.23 (0.90–1.67) | 1.32 (0.89–1.98) | 1.21 (0.88–1.65) | 0.99 (0.66-1.49) | 1.16 (0.85-1.59) |
| Suicide thoughts | 0.79 (0.50-1.24) | 1.51 (1.13-2.02) | 0.56 (0.35-0.89) | 1.44 (1.08-1.93) | 0.67 (0.42-1.08) | 1.37 (1.01-1.86) | 0.49 (0.31-0.80) | 1.31 (0.96-1.78) |
| Suicide attempts | 1.18 (0.58-2.39) | 2.40 (1.51-3.83) | 0.91 (0.45-1.85) | 2.31 (1.45-3.68) | 0.98 (0.48-2.00) | 2.03 (1.27-3.25) | 0.80 (0.39-1.65) | 1.97 (1.23-3.16) |
| At-risk alcohol use | 0.19 (0.11-0.33) | 0.69 (0.53-0.89) | 0.18 (0.10-0.31) | 0.68 (0.53-0.88) | 0.21 (0.12-0.37) | 0.68 (0.53–0.89) | 0.19 (0.11-0.34) | 0.68 (0.52–0.88) |
| Substance use | 0.66 (0.32-1.33) | 0.70 (0.40-1.20) | 0.31 (0.15-0.62) | 0.59 (0.33-1.03) | 0.62 (0.37-1.15) | 0.66 (0.37-1.15) | 0.31 (0.14-0.65) | 0.57 (0.32-1.02) |

**Fig A: Illustration of geographical origin (unweighted), time of conflicts, and age groups^1^.**

**
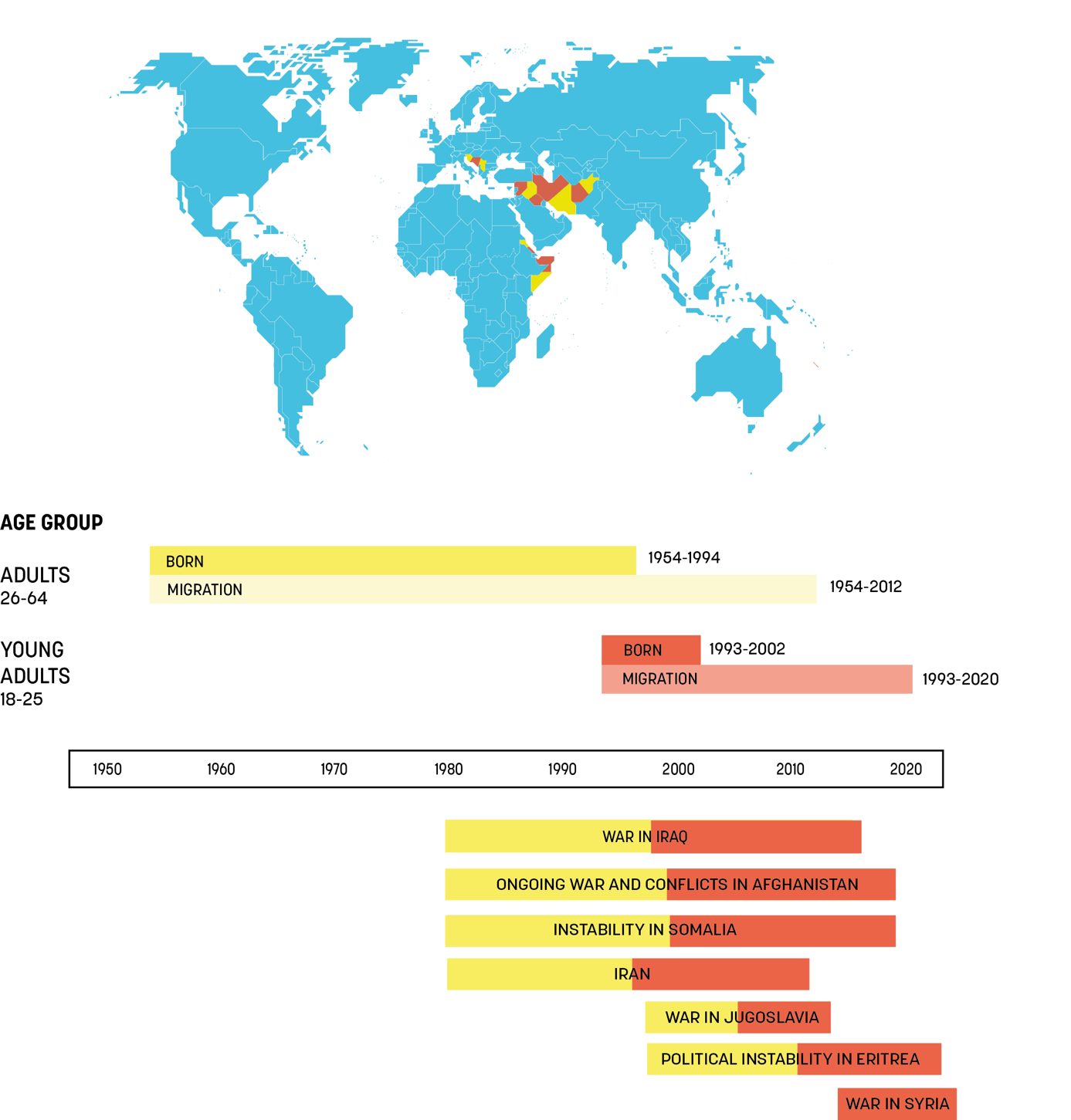
**

^1^Coloured areas represent the most common countries/areas of origin (unweighted).
