# Supplementary material for: Is refugee experience in childhood a risk for poorer health in adulthood?—A Swedish national survey study
Source: PLOS Glob Public Health. 2023 Nov 8;3(11):e0002433. doi: 10.1371/journal.pgph.0002433 (PMC10631682; doi:10.1371/journal.pgph.0002433)
Supplement: Attachment — Submitted filename: Response to Reviewers.docx [file pgph.0002433.s003.docx]

**Response to reviewer’s comments**

**Is refugee experience in childhood a risk for poorer health in adulthood? - A Swedish national survey study**

**Authors:**

**Erica Mattelin, Amal R. Khanolkar Laura Korhonen, Jill W. Åhs & Frida Fröberg,**

Reviewer #1: Please consider confounding by country and religion of origin for refugees. Alcohol use may be negatively associated with being muslim. Are the country of origin of migrants and refugees similar? Do the have differing distributions from different regions around the world that may account for differences in things like substance abuse, psychological distress?
We appreciate your insights regarding potential confounding factors related to country of origin and religion among refugees. As we used country of origin to create the exposure variable in our study (see Supplementary Table 1), it cannot be used as a confounder in our analysis.

Regarding religion, for confounding to occur, there are three conditions that need to be fulfilled. First, the confounding factor must be associated with both the risk factor of interest and the outcome. Second, the confounding factor must be distributed unequally among the groups being compared. Third, a confounder cannot be an intermediary step in the causal pathway from the exposure of interest to the outcome of interest.

Religion (here defined as relating to any religion) is an intermediary step between the exposure and the outcome in this case since refugees and migrants are more likely to be religious than those who are born in Sweden. This is because Sweden is one of the most secular countries in the world. Hence, religion does not fulfill condition number three for being a confounder.

Being a Muslim vs non-Muslim would imply the same problem. Being Muslim is unlikely the cause of migration, and hence the relationship would be that more refugees and migrants are Muslim (the arrow would move the same pathway as for religion in the directed acyclic graph (DAG) below) and hence not fulfill condition number three.

We have included a DAG below to provide further clarification on this point.

*Nonetheless information on religion was not collected in the survey.*

[References]

Greenland, S., Pearl, J., & Robins, J. M. (1999). Causal Diagrams for Epidemiologic Research. Epidemiology, 10(1), 37–48.

Jager, K. J., Zoccali, C., Macleod, A., & Dekker, F. W. (2008). Confounding: what it is and how to deal with it. Kidney international, 73(3), 256–260.

Westreich, D., & Greenland, S. (2013). The table 2 fallacy: presenting and interpreting confounder and modifier coefficients. American journal of epidemiology, 177(4), 292–298.

Williams, T.C., Bach, C.C., Matthiesen, N.B. et al. Directed acyclic graphs: a tool for causal studies in paediatrics. Pediatr Res 84, 487–493 (2018).

When you refer to multivariable regression are you referring to multinomial logistic regression? That is, are all of the groups in the same logistic regression model?
Thank you, multivariable regression is not the same as multinomial logistic regression. A multivariable regression model is one that contains multiple independent variables. We did not use multinomial logistic regression modeling in this study. We apologize for any confusion caused.

Please see: Statistical primer: multivariable regression considerations and pitfalls, SW Grant et at, 2018.

Also, are there any additional confounders that you can adjust for in those models?
Thank you for this comment. In terms of additional confounders, we do not believe there are any other relevant confounders included in our study that need to be adjusted for. You can refer to the DAG for more details on this.

Among the refugee women is it possible that they are unlikely to report suicidal thoughts? They appear to have a higher odds of suicidal attempts, but lower for suicidal thoughts. Could this be related to differences in how the questions are interpreted or comfort and reporting suicidal thoughts?
Thank you for this question. Regarding the lower odds of suicidal thoughts among refugee women compared to Swedish-born individuals, we agree that this is an interesting finding. While the odds for suicidal thoughts are indeed lower (OR 0.41, 95% CI 0.30-0.72), there is no significant association with suicidal attempts (OR 0.94, 95% CI 0.51-1.74). We would however like to interpret the causes of these numbers with caution.

Overall it could be that it cultural and religious status account for some of the differences in the self-reported metrics as opposed to immigrant or refugee status per say. It would help to differentiate that in the paper and consider sources of confounding.
We appreciate your overall observation that cultural and religious factors may account for some of the differences in self-reported metrics, rather than immigrant or refugee status alone. However, we would like to refer to the answer provided for the first question.

Reviewer #2: Is refugee experience in childhood a risk for poorer health in adulthood - A Swedish national survey study – PGPH-D-23-00525. The authors examined whether adults 18-64 years with a childhood refugee experience were more likely to self-rate their mental/general health as poor compared to non-refugee migrants and Swedish-born.
The manuscript presents rigorous original research findings that contribute to refugee studies and especially the long-term relationships between being a refugee and adult health/wellbeing. The authors performed technically sound inferential statistical analysis and described and discussed the findings in sufficient detail. Overall, the paper is well organized and clearly written. The data appears to support the conclusions drawn by the authors. However, the authors may focus on a few areas below to strengthen the manuscript.
Background
Your background is too short (less than 2 pages). There is much current literature regarding childhood refugee experiences and the risk of poor health in adulthood from a Swedish perspective. Kindly consider adding more detail to the background to provide a more compelling argument in which you would situate the need for your study.
Thank you for your positive feedback. We agree that there are some studies on childhood refugee experiences and health care utilization in Sweden. In the background, we have expanded on this topic and aimed to provide a better understanding of the complementary nature of our study (page 4-5, lines 101-119). Unlike the existing studies that are based on selective samples of individuals seeking healthcare services, our study utilizes data from a national public health survey, drawn from the general population. Moreover, while the other studies rely on clinicians' assessments of a selective population actively seeking healthcare, our study is based on self-assessment from the general population. These distinctions highlight the unique contribution and perspective of our research.

Methods and Materials
In your paper, it is not clear how you accounted for missing data. Kindly consider giving more details.
Thank you for raising this issue. We have clarified this further on page 6, line 165. Since the missing data was below <5% the likelihood of the estimates being biased is low and hence we conducted a complete case analysis.

[References]
*Derrick A. Bennett, How can I deal with missing data in my study? Australian and New Zealand Journal of Public Health, Volume 25, Issue 5, 2001, Pages 464-469.*

*Schafer J. L. (1999). Multiple imputation: a primer. Statistical methods in medical research, 8(1), 3–15. https://doi.org/10.1177/096228029900800102*

Refugees’ and immigrants' ethnicity and socioeconomic status are key in determining health and how they cope. I was expecting to see other key demographic variables such as participants’ employment status in Table 1. Kindly consider.
We appreciate your feedback. In our study, we have chosen to use education as a proxy for socioeconomic status, and this information is included in Table 1. However, if the Editor deems it necessary, we are open to including employment status in Table 1 as well.

Regarding the consideration of employment as a confounder in this analysis, we would like to clarify that employment status should not be considered a confounder in our study. In this case, employment status is an intermediary step between the exposure (refugee status) and the outcome, as refugee status can affect employment opportunities. Numerous studies, both nationally and internationally, have demonstrated both a more difficult and slower access to the labor market for migrants and refugees. Moreover, earlier studies have shown that factors such as the inability to access the labor market in the host country can contribute to mental ill-health in refugees and migrants. For further elaboration on confounders, we kindly refer you to our response to the first question raised by reviewer 1.

It is important to note that ethnicity is not measured in the survey we utilized, and therefore, we cannot incorporate it as a variable. Generally, data on ethnicity is not collected in Sweden. However, nationality is often used as a proxy for ethnicity, but since it is a part of the exposure variable it is impossible to use as a confounder.

[References]
Al-Zubaidi, Y. (2012). *Statistikens roll i arbetet mot diskriminering: en fråga om strategi och trovärdighet:[rapport*. Diskrimineringsombudsmannen.

Matlin, S.A., Depoux, A., Schütte, S. *et al.* Migrants’ and refugees’ health: towards an agenda of solutions. *Public Health Rev* **39**, 27 (2018). <https://doi.org/10.1186/s40985-018-0104-9>

Wikström, P. and Hübinette, T. (2021), Equality data as immoral race politics: A case study of liberal, colour-blind, and antiracialist opposition to equality data in Sweden. Br J Soc Psychol, 60: 1154-1176. https://doi.org/10.1111/bjso.12495

Åslund, O., Forslund, A., & Liljeberg, L. (2017). Flykting-och anhöriginvandrades etablering på den svenska arbetsmarknaden (No. 2017: 14). IFAU-Institute for Evaluation of Labour Market and Education Policy.

The Kessler Psychological Distress Scale measures a person’s psychological distress/emotional state within a period of 4 weeks. But you were seeking to measure the mental health of adults who migrated as refugees at a younger age. I wonder whether measuring the emotional state of your participants in the past 4 weeks only properly captured the psychological distress of the participants. The participants’ mental health might have been a cumulative result of events linked to being a refugee at a young age (which goes beyond 4 weeks). Why did the authors measure social distress only within the past 4 weeks and not beyond, such as the past 6 months, 12 months, etc.? Could this short period influence your results in any way? If so, could it be a limitation to how far you could interpret your findings? Kindly consider.
We acknowledge the limitations of using the Kessler-6 as a measure of mental health, as it is a brief screening tool and not a comprehensive diagnostic instrument. Ideally, distress would have been assessed using multiple time points and diagnostic interviews to provide a more detailed understanding of mental health. Unfortunately, as we were not involved in the design of the data collection, we did not have the opportunity to include such measures. However, it is worth noting that the Kessler-6 is widely utilized in several national public health surveys and has shown favorable performance compared to other similar measures. Its briefness makes it feasible for large-scale surveys, making it possible to collect mental health data on a broader population. We have addressed these considerations and expanded on the limitations of the Kessler-6 in the corresponding paragraph of the limitations section to provide a comprehensive assessment of its use in our study (pages 22-23)

[References]

*Furukawa, T. A., Kessler, R. C., Slade, T., & Andrews, G. (2003). The performance of the K6 and K10 screening scales for psychological distress in the Australian National Survey of Mental Health and Well-Being. Psychological medicine, 33(2), 357–362.* [*https://doi.org/10.1017/s0033291702006700*](https://doi.org/10.1017/s0033291702006700)

*Cornelius BL, Groothoff JW, van der Klink JJ, Brouwer S. The performance of the K10, K6 and GHQ-12 to screen for present state DSM-IV disorders among disability claimants. BMC Public Health. 2013 Feb 12;13:128. doi: 10.1186/1471-2458-13-128. PMID: 23402478; PMCID: PMC3575398.*

Discussion
Page 22 paragraph 3 line 2: ‘aren’t’, should be written in full as ‘are not’
Statements and Declarations
Page 23 paragraph 1 line 8: Check the sentence and change ‘However’ to ‘however’ appropriately.
Page 24 under ‘Availability of data and materials’, check the statement: ‘ However, data from the The Swedish National…’ and remove ‘The’ appropriately.
Thank you for pointing these errors out. All of these have been addressed.

Reviewer #3: Comments to the Author(s)

1. I believe a brief section on Swedish policies and programs for refugees and migrants would help contextualize the study findings and help readers appreciate the findings. For instance, such a section could brief describe the Swedish immigrants’ profile and current and historical policies on immigration.
Thank you for pointing this out, we have added this information in the introduction to give the reader some background on migration related policies in Sweden (page 5 (lines 120-131).

2. Even though I appreciate the effort and creativity of the author(s) in creating their variable, I wonder if “Those with refugee experience in childhood (0-17 years)” adequately captures the refugee experience. Would older children better capture the refugee experience, say 10-17 than 0-17? I am particularly concerned that 0-5year olds may not remember the refugee experience and may have similar experience to those who migrated in childhood for reasons not associated with refugee status or asylum seeking.

Thank you for your suggestion. We appreciate the interesting discussion. However, we want to emphasize that we do not conceptualize the refugee experience as ending upon arrival in Sweden. The refugee experience is likely ongoing, characterized by post-migratory stressors and potential impacts of parental distress. Therefore, even individuals who arrived between the ages of 0 and 5 would continue to have experiences associated with being a refugee. However, we agree that their exposure to adverse events, such as violence, before and during migration may differ from those who arrived at a later age, for example, at 17 years. The timing of these different exposures is intriguing and warrants further investigation in future research.

Regarding your suggestion, we conducted a sensitivity analysis to assess if our results would significantly change by excluding individuals under the age of 5 and under the age of 10. We found no major changes, except for general health in refugee women. Additionally, the sample size reduction resulted in wider confidence intervals for some outcomes.

When excluding the 0-5-year-olds from the analysis, the only notable change is that the confidence intervals for suicide attempts and general health become wider for migrants and are no longer statistically significant. However, for women, the results for drug use went from non-significant to significant, with an odds ratio of 0.29 (0.11-0.77).

When excluding the 0-10-year-olds, the results for drug use in refugee women become significant, with an odds ratio of 0.13 (0.03-0.68). However, the differences in psychological distress among migrant women are no longer statistically significant, with an odds ratio of 1.28 (0.88-1.86).

The only result that significantly changes direction is general health in refugee women. The odds ratio shifts from 1.15 (0.62-2.12) to 0.48 (0.23-0.99).

We are happy to include these results as a supplementary table if the Editor wishes us to.

3. Further, it would have love to see the authors extend the control variables to include socio-economic variables such as household income, education, religion etc. if these are available in the Swedish National Public Health Survey..
Thank you for this comment. For a more elaborate answer please see our answer to question 1, review 1, and question 3, reviewer 2.

4. Even though the authors acknowledged and addressed the change in instruments used to assess psychological distress, I wonder how the differences in the recall period between the previous two weeks of the GHQ-5 and the past four weeks with (Kessler-6) might affect the results.
We appreciate your comment. We acknowledge that the change in the instrument used for measuring psychological distress and symptoms is a limitation, but it was beyond our control. It is worth noting that several studies demonstrate the superiority of the Kessler-6 (K6) scale compared to the General Health Questionnaire (GHQ) which is most likely the reason for the change in the instrument between 2018 and 2020. While we believe that the K6 performs better, we recognize that it would have been preferable to maintain consistency in the measurement instrument throughout the study.

Regarding the concern for the recall period, we have addressed this issue in the discussion of the paper (pages 22-23), where we discuss the potential impact of recall bias on the validity of our findings.

Furthermore, we want to highlight that we have previously published an article utilizing the same dataset and methodology (Mattelin E. et al., Lancet Eclinical Medicine, 2022) that went through rigorous peer review (Lancet Public Health and Lancet eClinical Medicine).

[References]

*Furukawa, T. A., Kessler, R. C., Slade, T., & Andrews, G. (2003). The performance of the K6 and K10 screening scales for psychological distress in the Australian National Survey of Mental Health and Well-Being. Psychological medicine, 33(2), 357–362.* [*https://doi.org/10.1017/s0033291702006700*](https://doi.org/10.1017/s0033291702006700)

*Cornelius BL, Groothoff JW, van der Klink JJ, Brouwer S. The performance of the K10, K6 and GHQ-12 to screen for present state DSM-IV disorders among disability claimants. BMC Public Health. 2013 Feb 12;13:128. doi: 10.1186/1471-2458-13-128. PMID: 23402478; PMCID: PMC3575398.*

*Mattelin, E. Fröberg, F., Korhonen, L & Khanolkar, A. (2022) Health and health-related behaviours in refugees and migrants who self-identify as sexual or gender minority – A National population-based study in Sweden*

*eClinicalMedicine, Volume 52, 101641*

5. I suggest the authors provide the distribution of their outcome variables and briefly indicate what percentage for instance were psychological distressed, and how did that vary by status in Sweden.
Thank you for this comment. We might misunderstand what the reviewer is asking for, but the percentages are presented in Table 2 on page 13.

6. How did the authors results on psychological distress compare with other countries that used a similar instrument. The supplementary file did not seem to contain the distribution of the outcome variables.
Thank you for this comment. The distribution of the outcome variable is presented in Table 2. We do not know of any comparable study that has examined this research question using similar data and more importantly also including non-refugee migrants as a comparator group. This limits comparisons.

Regarding the comparison to other studies, we found that the prevalence of psychological distress in our study is lower than that reported in a US study from 2018, which utilized the K6 measure (22.6% in the US study). In our study, the prevalence differs between groups. But is for example 10.4 for Swedish-born males and 21.4 for females with a refugee experience in childhood. Similarly, a Canadian study using the K10 measure found comparable results, with a prevalence of high psychological distress at 20.74% in the general population. However, when compared to a similar survey conducted in Finland, our study's numbers are higher. In the Finnish study, women reported higher psychological distress rates compared to men, with prevalence rates of 11.0% and 8.8% respectively. It's important to note that the Finnish study used a different instrument to measure psychological distress.

In summary, our study's findings suggest a lower prevalence of psychological distress compared to the US and Canadian studies that utilized similar measures. However, when compared to the Finnish study, our study indicates higher levels of psychological distress. We have included a paragraph on this on page 23.

[References]

Caron, J., Liu, A. Factors Associated with Psychological Distress in the Canadian Population: A Comparison of Low-Income and Non-Low-Income Sub-Groups. *Community Ment Health J* **47**, 318–330 (2011). <https://doi.org/10.1007/s10597-010-9306-4>

Daly, M. Prevalence of Psychological Distress Among Working-Age Adults in the United States, 1999–2018”, American Journal of Public Health 112, no. 7 (July 1, 2022): pp. 1045-1049.

Viertiö, S., Kiviruusu, O., Piirtola, M. *et al.* Factors contributing to psychological distress in the working population, with a special reference to gender difference. *BMC Public Health* **21**, 611 (2021). <https://doi.org/10.1186/s12889-021-10560-y>

7. On page, 17, the authors indicated that among adults, being a refugee was associated with lower odds of at-risk alcohol use than Swedish-born. Could this be of a result of not controlling for or accounting for the religious and cultural affiliations of immigrants?
Thank you for this comment. Regarding religion, we would like to refer to answer 1, reviewer 1.

Although we acknowledge the potential significance of examining country/region of origin, we were unable to include this variable as a confounder in our models. Country of origin was used as part of developing our exposure of interest. Nevertheless, our findings align with a nationwide cohort study that similarly indicated a reduced risk of alcohol use. It is important to note, however, that studies have highlighted differences in alcohol use among individuals from different regions of origin. It is also worth noting that none of these studies specifically focused on self-reported alcohol use among individuals who migrated to Sweden as children.

[References]

Harris S, Dykxhoorn J, Hollander AC, Dalman C, Kirkbride JB (2019) Substance use disorders in refugee and migrant groups in Sweden: A nationwide cohort study of 1.2 million people. PLOS Medicine 16(11): e1002944. <https://doi.org/10.1371/journal.pmed.1002944>

Hjern, A. and Allebeck, P. (2004), Alcohol-related disorders in first- and second-generation immigrants in Sweden: a national cohort study. Addiction, 99: 229-236. <https://doi.org/10.1046/j.1360-0443.2003.00562.x>

8. I believe the authors could strengthen the discussion by indicating the potential policy implications of their study for Sweden and other high-income countries that accept migrants.
Thank you for pointing out that out. We have tried to clarify the policy implications of our findings on pages 24-25.

9. The authors indicated on page 22 that the surveys were only distributed in three languages, did this limit potential participation?
We appreciate your comment. It is important to note that all participants included in our study arrived *before the age of 18,* which implies that they would have been integrated into the Swedish school system and/or entitled to free Swedish language education provided to immigrants. Therefore, language proficiency should not significantly limit participation in the study. Moreover, Statistics Sweden has applied weights to account for non-response, which helps to address potential biases and adjust for different response patterns.

Further, we have made edits to improve grammar and readability throughout the manuscript.
