# Supplementary material for: Is refugee experience in childhood a risk for poorer health in adulthood?—A Swedish national survey study
Source: PLOS Glob Public Health. 2023 Nov 8;3(11):e0002433. doi: 10.1371/journal.pgph.0002433 (PMC10631682; doi:10.1371/journal.pgph.0002433)
Supplement: Attachment — Submitted filename: Response letter_Mattelin.docx [file pgph.0002433.s004.docx]

**Response to reviewer’s comments**

**Is refugee experience in childhood a risk for poorer health in adulthood - A Swedish national survey study**

**Authors:**

**Erica Mattelin, Amal R. Khanolkar Laura Korhonen, Jill W. Åhs & Frida Fröberg,**

Reviewer #3: Comments to the authors

I commend the authors for addressing most of my comments and suggestions satisfactorily. However, there are a few minor comments on the updated manuscript. These comments are shown below:

The authors on page 4 indicated that “Many refugee children also experience mental health issues during the first few years after arrival in a host country. Refugee children have a higher prevalence of PTSD, depression, and anxiety disorders shortly after resettlement in a host country than native-born peers (7). In general, a childhood refugee experience was not associated with worse self-rated health or risk behaviours in adulthood when considering age and sexual- and gender-minority status”. It might be useful to provide a broader context and indicate the systemic factors in Sweden that often lead to these experiences.

It might also be important to contextualize the key finding of the study that “In general, a childhood refugee experience was not associated with worse self-rated health or risk behaviours in adulthood when considering age and sexual- and gender-minority status”. This might be due to the various protective programs offered by Swedish immigration policy.

In the first paragraph of the discussion section, it might be useful for the authors to provide some context and provide potential reasons for their findings. The authors attempted doing this at the later part of the strengths and limitations section on page 23. It might be relevant to move those parts to after the first paragraph of the discussion section.

Thank you for these comments. We agree with the reviewer and find that it improved the manuscript to earlier describe potential explanations for our findings. We have included a further elaboration in the introduction on the Swedish system (page 5, lines 131-135) and we have also made the suggested change to move relevant explanations from the "Strengths and Limitations" section to the first paragraph in the discussion (page 20) to provide better context for our findings. Hopefully, this makes it clearer.

There a few typographical errors and the manuscript might benefit from another round of review. For instance, on in the abstract lines 54 and 55, it reads: were analysed using logistic regression, adjusted for age and sexual- and gender-minority (SGM) identity, and run stratified by sex”

Thank you for this comment. We have reviewed the manuscript again and made minor typographical and grammatical changes throughout, including correcting the mentioned errors in the abstract. Hopefully, this has improved clarity.
